# Qualitative and quantitative CT analyses of the solid component in lung adenocarcinoma for predicting invasiveness

**DOI:** 10.1007/s11604-025-01794-6

**Published:** 2025-05-25

**Authors:** Yoshie Kunihiro, Fumi Kameda, Taiga Kobayashi, Masahiro Tanabe, Ryoko Morooka, Toshiki Tanaka, Yoshinobu Hoshii, Tsuneo Matsumoto, Katsuyoshi Ito

**Affiliations:** 1 Department of Radiology, Yamaguchi University Graduate School of Medicine, 1-1-1Minamikogushi, Ube, Yamaguchi 755-8505 Japan; 2 Department of Radiology, Ube Central Hospital, 750 Nishikiwa, Ube, Yamaguchi 755-0151 Japan; 3 Department of Surgery and Clinical Science, Division of Chest Surgery, Yamaguchi University Graduate School of Medicine, 1-1-1Minamikogushi, Ube, Yamaguchi 755-8505 Japan; 4 Department of Diagnostic Pathology, Yamaguchi University Hospital, 1-1-1Minamikogushi, Ube, Yamaguchi 755-8505 Japan; 5 Department of Radiology, National Hospital Organization Yamaguchi - Ube Medical Center, 685 Higashikiwa, Ube, Yamaguchi 755-0241 Japan

**Keywords:** X-ray computed tomography, Lung adenocarcinoma, Quantitative analysis, Multivariate analysis

## Abstract

**Purpose:**

This study aimed to evaluate the CT findings of lung adenocarcinoma with solid components and to determine the difference between adenocarcinoma in situ (AIS) and minimally invasive adenocarcinoma (MIA) with invasive adenocarcinoma (IAC).

**Materials and methods:**

A total of 54 cases were included in this study. The diagnoses of lung adenocarcinoma consisted of AIS or MIA (*n* = 20) and IAC (*n* = 34). The following factors were evaluated on CT images: part-solid nodule or solid nodule, presence of air bronchogram, air space, calcification within the tumor, presence of interstitial pneumonia and emphysema, diameters of the tumor and solid component, and CT values of the solid component. The volume and CT number histograms, including the 50th, 75th, and 100th percentiles of solid component were obtained using a software program. The CT criteria were compared between AIS, MIA, and IAC, and an indicator of differentiation was considered.

**Results:**

Part-solid nodules were observed more frequently in AIS and MIA (85.0%) than in IAC (55.9%). All criteria for quantitative analysis showed significant differences between AIS or MIA and IAC, and the diameter of the solid component in the mediastinal window was an indicator of differentiation (*p* = 0.0006; odds ratio, 1.4; 95% confidence interval, 1.2–1.8).

**Conclusion:**

The diameter of the solid component on the mediastinal window was considered an indicator of differentiation between AIS, MIA, and IAC.

Condensed abstract.

Quantitative data of solid component, including both manual measurements and evaluation using CT software, are correlated with pathological invasiveness.

Diameter of the solid component in the mediastinal window would be an indicator of IAC.

## Introduction

The invasiveness of lung adenocarcinoma is described in the International Association for the Study of Lung Cancer/American Thoracic Society/European Respiratory Society multidisciplinary classification; adenocarcinoma in situ (AIS) is a localized ≤ 3 cm adenocarcinoma lacking stromal, vascular, or pleural invasion; minimally invasive adenocarcinoma (MIA) is a localized ≤ 3 cm adenocarcinoma with ≤ 0.5 cm invasion; and invasive adenocarcinoma (IAC) is an adenocarcinoma with ≥ 0.5 cm invasion [1]. Radiological staging based on the 8 th lung cancer TNM classification depends on the presence and size of the solid component, and there are proposals associated with CT evaluation for the invasiveness of lung adenocarcinoma, which typically shows a pure ground-glass nodule (GGN), MIA usually shows a ground-glass predominant nodule with *a* ≤ 0.5 cm solid component, and IAC usually shows a nodule with *a* ≥ 0.5 cm solid component [2]. Previous studies associated with CT evaluation showed that pure GGN could be invasive, and that larger size, high CT attenuation, heterogeneous density, irregular shape, and vessel changes were useful for predicting invasiveness [3–12]. The solid component could correspond to stromal or vascular invasion, collapsed alveolar space, fibroblastic proliferation, infiltration, and mucin [13]. AIS could have a solid component on CT due to alveolar collapse [9, 14–16]. However, the sizes and findings of solid components on CT images that are useful for predicting pathologic invasiveness are still controversial, and CT analyses focused on solid components have not been well evaluated.

The purpose of this study was to evaluate the CT images of lung adenocarcinoma with solid components and to determine an indicator for differentiating between AIS or MIA and IAC.

## Materials and methods

This study was approved by the institutional review board of our institution. The requirement for informed consent was waived due to the retrospective study design.

### Patients

Medical records and CT images were obtained at our institution between January 2017 and December 2021. The inclusion criteria were as follows: (a) patients who were diagnosed with lung adenocarcinoma by surgical resection; (b) preoperative CT images acquired within 1 month before surgery; (c) nodules with solid components that can be evaluated on CT in both lung and mediastinal window settings. We found 180 cases who met inclusion criteria. The exclusion criteria were as follows: (a) history of lung resection, chemotherapy, or radiation therapy for thoracic carcinoma (*n* = 29); (b) pT2-T4 lung carcinoma (*n* = 95). In addition, 2 cases were excluded, because they involved pulmonary infection and interstitial pneumonia, respectively, and the CT findings had not been evaluated correctly. A total of 54 cases (male, *n* = 28; female, *n* = 26; age [mean ± standard deviation], 71.8 ± 8.5 years; age range, 37–82 years) were included. Two patients had 2 lung adenocarcinomas. The diagnoses of lung adenocarcinoma consisted of AIS or MIA (*n* = 20: AIS, *n* = 12; MIA, *n* = 8) and IAC (*n* = 34). One pathologist evaluated the pathological invasive size for this study. The IAC group consisted of 11 acinar, 8 papillary, 6 lepidic, 5 invasive mucinous, and 4 solid adenocarcinomas. Pathological stages were determined based on the criteria of the International Union Against Cancer 8.

### CT examinations

All chest CT scans were obtained using a 64-row detector CT scanner (SOMATOM FORCE; Siemens) with a slice thickness of 1 mm without a gap. The scans were obtained at suspended end inspiration effort in the supine position without intravenous contrast medium injection. The scanning parameters were 100 kVp and automated mA. The CT images were reconstructed with hybrid-iterative reconstruction (Hybrid-IR) with Siemens ADMIRE. All image data were interfaced directly with the picture archiving and communication system (PACS) (ShadeQuest; Yokogawa Medical Solutions Corp.), and monitors were used to view the CT image (window width (WW), 1500 HU; window level (WL), − 600 HU at lung settings and WW, 255 HU; WL, 50 HU at mediastinal settings).

### CT image analysis

Two thoracic radiologists (Y. K. and F. K., with 20 and 10 years of experience, respectively) independently evaluated the CT images. Discordant results between the two radiologists were resolved by consensus. The following CT findings were coded: (a) part-solid or solid nodule, (b) air bronchogram, (c) air space, (d) calcification, (e) interstitial pneumonia, and (f) emphysema. The criteria of interstitial pneumonia in this study include the findings of interstitial lung abnormalities; ground-glass opacity and reticular opacity with or without traction bronchiectasis and honeycombing, which affect more than 5% of the upper, middle, or lower lung zone, as demarcated by the levels of the inferior part of the aortic arch and the right inferior pulmonary vein, respectively [17, 18]. The longest diameters of the entire tumor at the lung window setting (g), solid components in both lung (h) and mediastinal (i) window settings, and mean, maximum, and minimum CT values within the solid component at the mediastinal window setting (j) were measured on any one of axial, sagittal, and coronal CT images by the two chest radiologists. The mean value was then noted. For (i), the following two settings were also evaluated: WW, 1085; WL, −380 and WW, 670; WL, −165 to determine the window setting which correlate most closely with pathological invasiveness.

**Fig. 1:**
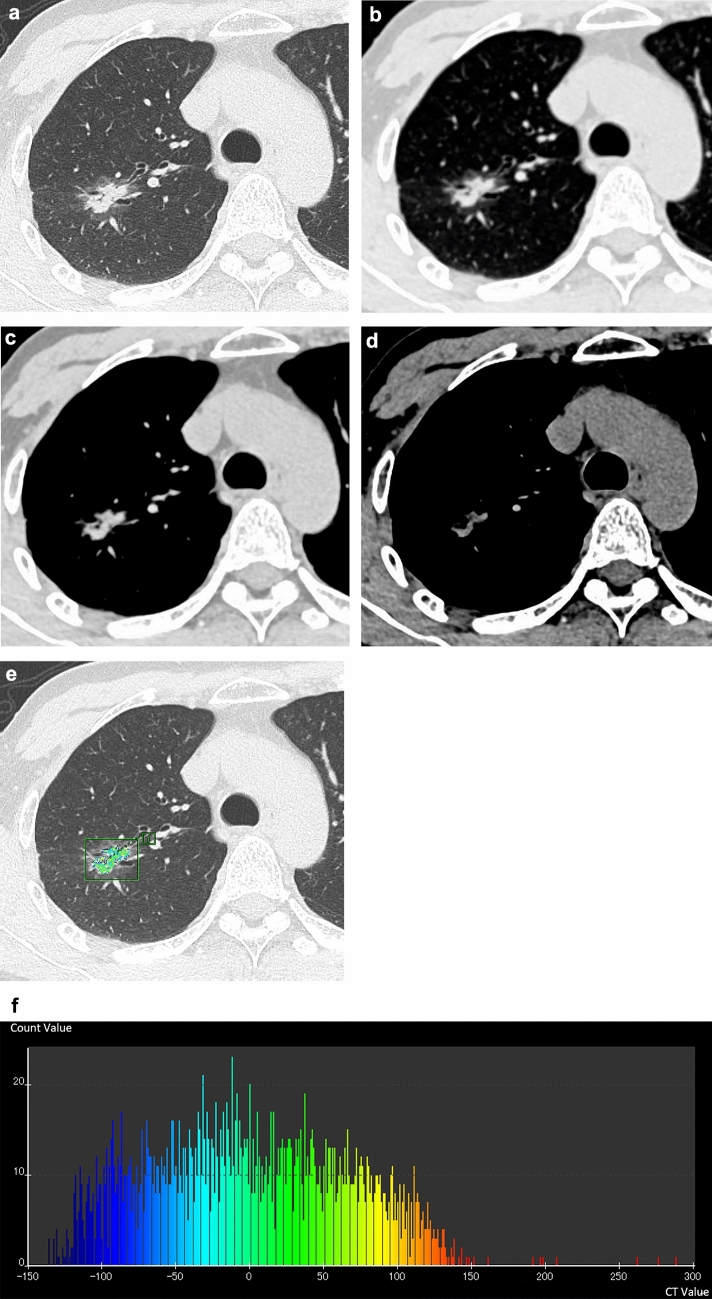
(**A–D**) A 59-year-old man with invasive adenocarcinoma. HRCT shows a part-solid nodule and the solid component is also observed in the mediastinal window setting. Diameter of the entire tumor = 2.7 cm, solid component = 2.06 cm (**A**), 1.93 cm (**B**), 1.91 cm (**C**), and 1.58 cm (**D**). The mean CT value of the solid component was 45.5 HU. (**E, F**) The volumes, and the 50 th, 70 th, and 100 th percentiles of the solid component were 0.9 cm^3^, −4 HU, 51 HU, and 288 HU, respectively

For (j), the solid component without calcification was evaluated manually. In addition, the volume and CT number histograms, including the 50 th, 75 th, and 100 th percentiles of the solid component were automatically obtained using a software program (AZE VirtualPlace3.8001) (Fig. 1).

### Statistical analysis

Sex, smoking history, tumor location, pathological stage, and qualitative CT findings were compared between AIS or MIA and IAC using a Chi-square (χ2) test for independence and Fisher’s exact probability test, if required. The age and quantitative analysis data of the CT images were compared using the Mann–Whitney test. The quantitative analysis data on CT and pathological invasiveness size measured by a single pathologist were compared using Spearman’s correlation analysis. In the next step, multiple logistic regression analyses were conducted to identify the significant indicators for differentiating between AIS, MIA, and IAC. A receiver-operating characteristic (ROC) curve analysis was used to determine the optimal cut-off values of the continuous variables; the area under the curve (AUC) was calculated. *P* values of < 0.05 were considered to indicate statistical significance. The forward selection (likelihood ratio) method was used for multiple logistic regression analysis. To reduce the effect of compounding factors, the longest diameters of solid components in the mediastinal settings (WW, 255 HU; WL, 50 HU) for (i), and the 100 th percentiles (histogram) of the solid component were selected. All other variables, including the parametric factors, were included. Interobserver agreement between the two radiologists was calculated as the kappa value (κ) for the aforementioned CT findings from (a) to (f) and as the intraclass correlation coefficient (ICC) for the measurements of CT images from (g) to (j) rated as follows: slight (0.00–0.20), fair (0.21–0.40), moderate (0.41–0.60), substantial (0.61–0.80), or almost perfect (0.81–1.00). The ICC was assessed using SPSS (version 22.0, IBM), and all other statistical analyses were performed using the JMP® Pro 15 software program (SAS Institute Inc.).

## Results

Patient characteristics are shown in Table 1. There were no significant differences in the clinical characteristics *(p* > 0.05), except for the pathological stage (*p* < 0.0001).

**Table 1:** The characteristics of patients with lung adenocarcinoma

		AIS or MIA (*n*=20)	IAC (*n*=34)	*p*-value
Age*		72.4 ± 7.17	71.4 ± 9.45	0.6969
Male, *n* (%)		10 (50.0)	18 (52.9)	0.8345
Smoking history, *n* (%)		12 (60.0)	18 (52.9)	0.6142
Tumor location, *n* (%)	Right upper lobe	5 (25.0)	14 (41.1)	0.7501
	Right middle lobe	3 (15.0)	3 (8.82)	
	Right lower lobe	2 (10.0)	4 (11.8)	
	Left upper lobe	5 (25.0)	7 (20.6)	
	Left lower lobe	5 (25.0)	6 (17.6)	
Pathological stage, *n* (%)	0	12 (60.0)	0 (0.00)	<0.0001
	I	8 (40.0)	29 (85.3)	
	II	0 (0.00)	4 (11.8)	
	III	0 (0.00)	1 (2.94)	

*AIS* Adenocarcinoma in situ, *MIA* Minimally invasive adenocarcinoma, *IAC* Invasive adenocarcinoma

*mean ± standard deviation

The qualitative and quantitative data are presented in Table 2. Part-solid nodules were significantly more frequent in AIS or MIA (85.0%) than in IAC (55.9%), and solid nodules were significantly more frequent in IAC (44.1%) than in AIS or MIA (15.0%) (*p* = 0.0284); however, there were no significant differences in other CT findings for qualitative criteria (presence of air bronchogram, air space, calcification within solid component, interstitial pneumonia, and emphysema) (*p* > 0.05).

**Table 2:** Results of the qualitative and quantitative CT findings with lung adenocarcinoma

CT analysis			AIS or MIA (*n*=20)	IAC (*n*=34)	*p*-value	Interobserver Agreement
Morphology	Part-solid nodule, *n* (%)		17 (85.0)	19 (55.9)	0.0284	0.78
	Solid nodule, *n* (%)		3 (15.0)	15 (44.1)		
Within solid component	Air bronchogram, n (%)		15 (75.0)	24 (70.1)	0.7267	0.54
	Cavity, *n* (%)		1 (5.00)	5 (14.7)	0.2731	0.78
	Calcification, *n* (%)		1 (5.00)	1 (2.94)	0.6989	0.66
Interstitial pneumonia, *n* (%)			4 (20.0)	8 (23.5)	0.7632	0.61
Emphysema, *n* (%)			4 (20.0)	7 (20.1)	0.9587	0.76
Diameter of tumor on CT (cm)			1.83 (0.91)	2.27 (1.26)	0.0411	0.88
Diameter of solid component of tumor on CT (cm)	Lung window	WL=−600, WW=1500	0.92 (0.46)	1.70 (1.01)	<0.0001	0.87
	Mediastinal window	WL=−380, WW=1085	0.87 (0.49)	1.67 (0.98)	<0.0001	0.91
		WL=−165, WW=670	0.83 (0.50)	1.63 (0.99)	<0.0001	0.92
		WL=50, WW=255	0.46 (0.49)	1.41 (1.02)	<0.0001	0.90
CT value of solid component (HU)		Mean	24.6 (16.4)	34.1 (19.2)	0.0229	0.60
		Maximum	47.3 (36.5)	69.3 (18.6)	0.0015	0.81
		Minimum	2.50 (39.3)	15.0 (18.8)	0.0282	0.27
Software analysis of solid component	Volume (cm^3^)		0.19 (0.31)	1.04 (1.16)	<0.0001	
	50^th^ percentile (HU)		−34.0 (100.3)	5.50 (26.0)	0.0016	
	75^th^ percentile (HU)		23.0 (95.3)	53.5 (52.9)	0.0005	
	100^th^ percentile (HU)		127.5 (142.3)	241.0 (189.0)	0.0006	

Data are presented as medians, with interquartile ranges in parentheses

*CT* Computed tomography, *AIS* Adenocarcinoma in situ, *MIA* Minimally invasive adenocarcinoma, *IAC* Invasive adenocarcinoma, *WL* Window level (HU), *WW* Window width (HU)

There were significant differences in all measurements (the longest diameter of the entire tumor at the lung window setting, solid component in both lung and mediastinal window settings, mean CT value within solid component at the mediastinal window setting, and volume, the 50 th, 75 th, and 100 th percentiles of solid component) (*p* < 0.05); all values were significantly larger in IAC than in AIS or MIA. Interobserver agreement was moderate to almost perfect (*κ* = 0.54–0.78 and ICC = 0.60–0.92) except for the minimum CT value (ICC = 0.27, indicating fair agreement).

**Fig. 2:**
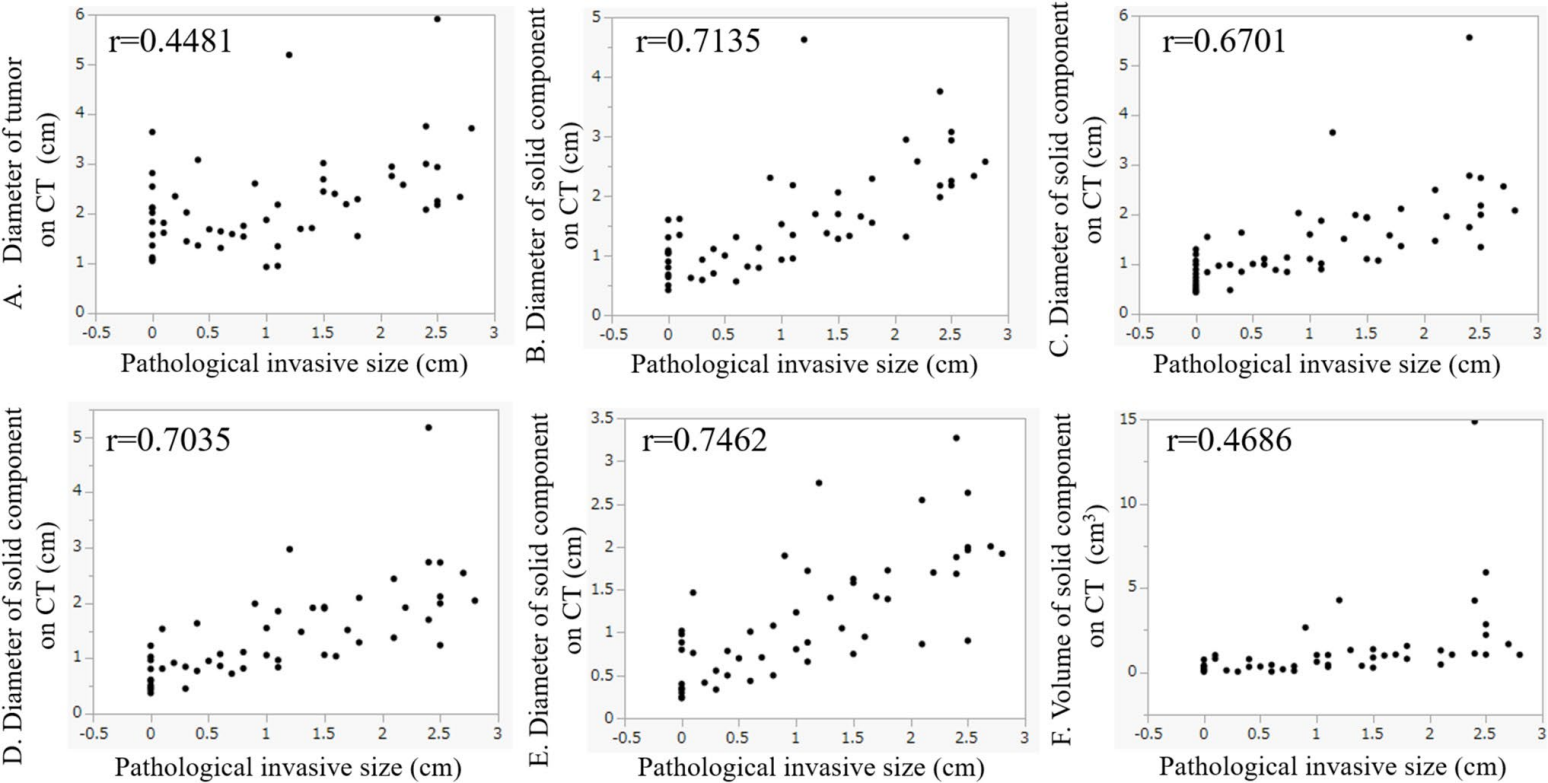
Correlation between the pathological invasive size and the diameter and volume of the tumor on CT. The correlation coefficient of each score was as follows: The correlation between the pathological invasive size and **A** the diameter of the tumor on CT in the lung window setting (*r* = 0.4481), **B** the diameter of the solid component on CT in the lung window setting (*r* = 0.7135), **C** the diameter of the solid component on CT in the mediastinal window setting (WL = −380, WW = 1085) (*r* = 0.6701), **D** the diameter of the solid component on CT in the mediastinal window setting (WL = −165, WW = 670) (*r* = 0.7035), **E** the diameter of the solid component on CT in the mediastinal window setting (WL = 50, WW = 255) (*r* = 0.7462), and **F** the volume of the solid component on CT (*r* = 0.4686)

**Fig. 3:**
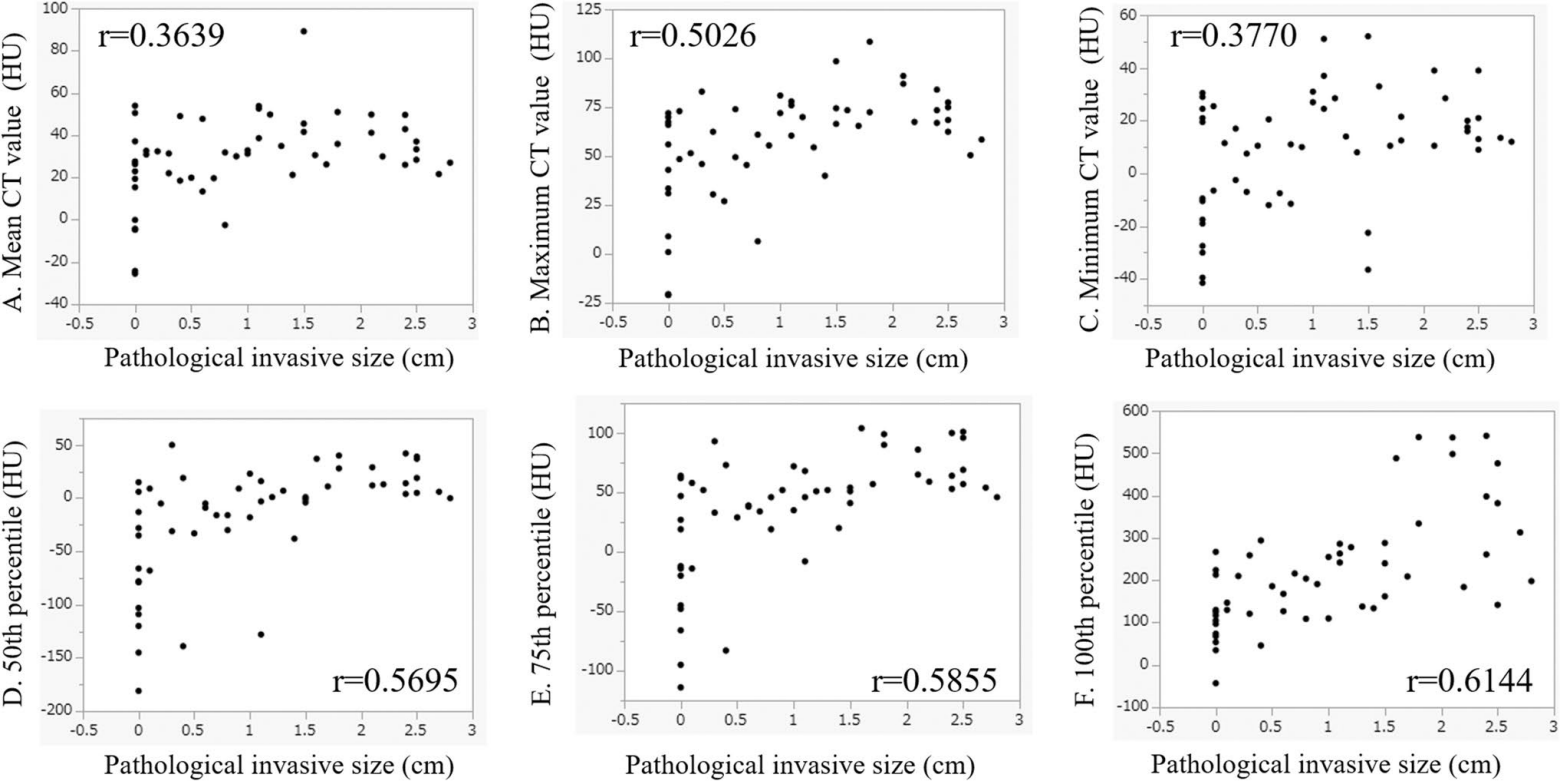
The correlation between the pathological invasive size and CT values and the data of the histogram analysis. The correlation coefficients for each score were as follows: Correlation between pathological invasive size and **A** mean CT value (HU) (*r* = 0.3639), **B** maximum CT value (*r* = 0.5026), **C** minimum CT value (*r* = 0.3770), **D** 50 th percentile (*r* = 0.5695), **E** 75 th percentile (*r* = 0.5855), and **F** 100 th percentile (*r* = 0.6144)

Spearman's correlation analysis showed a fairly strong correlation between the diameter of the solid component on CT in both lung and mediastinal window settings and pathological invasiveness size (*r* = 0.67–0.75). There were moderate correlations between the diameter of the tumor (*r* = 0.45), tumor volume (*r* = 0.47), and pathological invasiveness (Fig. 2). There were weak-to-fairly strong correlations between other quantitative analysis data, including the CT values and the histogram analysis of the solid component and pathological invasiveness size (*r* = 0.36–0.61, Fig. 3). The correlation coefficient of the mediastinal window setting (WW: 255 HU; WL: 50 HU) was the largest (*r* = 0.75).

**Fig. 4:**
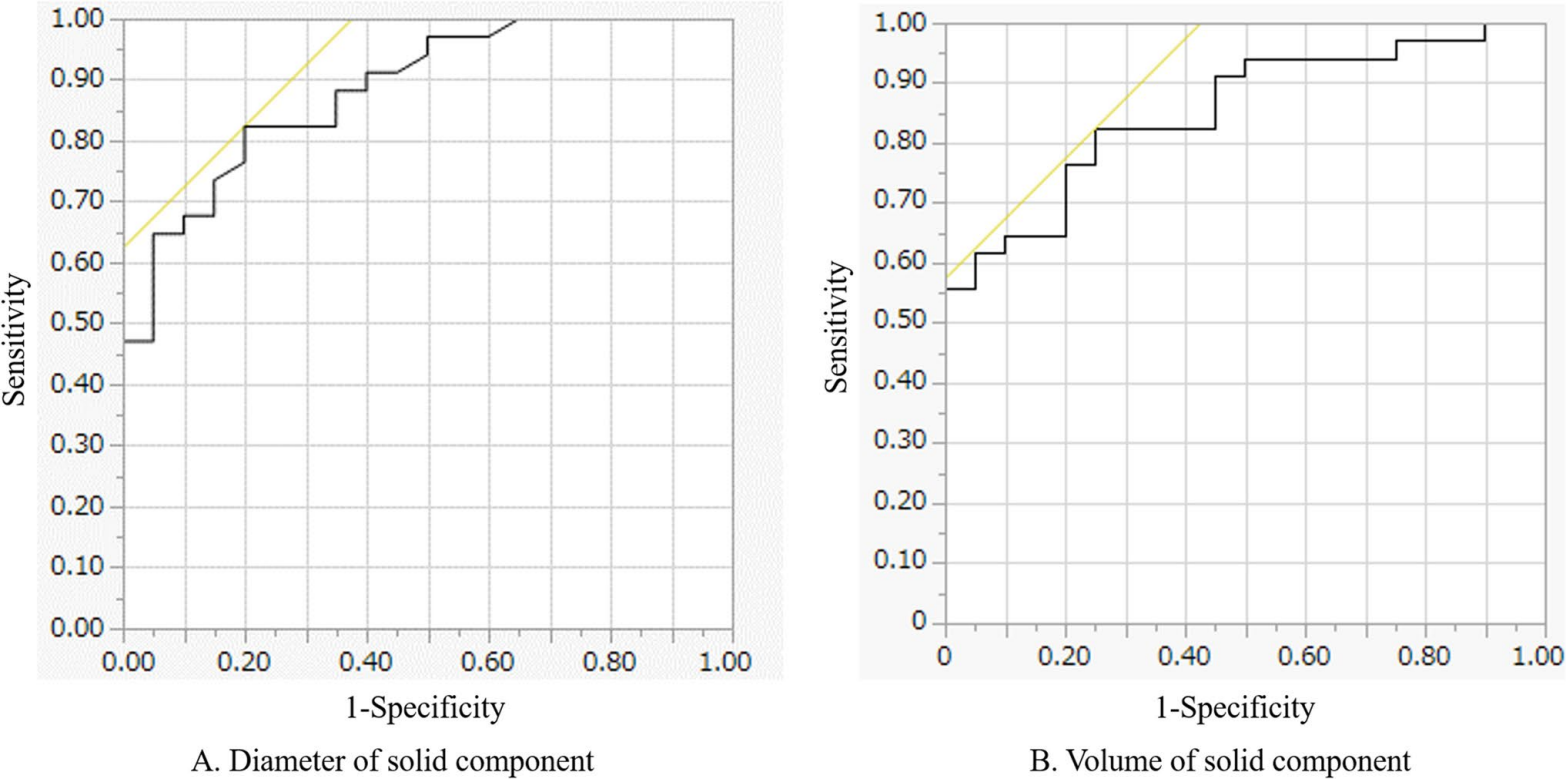
The area under the receiver-operating characteristic curve (AUC) of the diameter of the solid component on the mediastinal window setting (**A**) and the volume of the solid component (**B**). Based on predicting invasive adenocarcinoma. **A **The cut-off value was determined to be 0.80 cm (sensitivity = 82.6%, specificity = 80.0%, and AUC = 0.88). **B** The cut-off value was determined to be 0.37 cm.^3^ (sensitivity = 82.4%, specificity = 75.0%, and AUC = 0.85)

Multiple logistic regression analyses identified the diameter of the solid component in the mediastinal window (WW, 255 HU; WL, 50 HU) as an indicator of differentiation between AIS or MIA and IAC (*p* = 0.0006; odds ratio, 1.4; 95% confidence interval, 1.2–1.8). Table 3 shows the cut-off values obtained using quantitative analysis for predicting IAC. The cut-off value of the diameter of the solid component in the mediastinal window setting (WW, 255 HU; WL, 50 HU) for predicting IAC was determined to be 0.8 cm (sensitivity = 82.6% and specificity = 80.0%). The AUC was 0.88 (Fig. 4). With the exception of the diameter of the solid component on CT, the AUC for the volume of the solid component was also relatively high (0.85). The cut-off value of the solid component was 0.37 cm^3^ (sensitivity = 82.4% and specificity = 75.0%) (Fig. 4).

**Table 3:** Cutoff values using the quantitative analysis for predicting IAC

CT analysis			Cutoff value	Sensitivity (%)	Specificity (%)	AUC
Diameter of tumor on CT (cm)			2.18	61.8	75.0	0.668
Diameter of solid component of tumor on CT (cm)	Lung window	WL=−600, WW=1500	1.13	82.4	80.0	0.847
	Mediastinal window	WL=−380, WW=1085	1.08	82.4	80.0	0.876
		WL=−165, WW=670	1.06	79.4	85.0	0.876
		WL=50, WW=255	0.80	82.4	80.0	0.883
CT value of solid component (HU)		Mean	32.8	55.9	80.0	0.687
		Maximum	49.5	88.2	55.0	0.762
		Minimum	8.00	82.4	55.0	0.681
Software analysis of solid component	Volume (cm^3^)		0.37	82.4	75.0	0.853
	50^th^ percentile (HU)		−18.0	88.2	65.0	0.759
	75^th^ percentile (HU)		34.0	88.2	65.0	0.749
	100^th^ percentile (HU)		134.0	88.2	60.0	0.784

*CT* Computed tomography, *IAC* Invasive adenocarcinoma, *WL* Window level (HU), *WW* Window width (HU)

## Discussion

A solid component on CT tends to indicate an invasive lesion in lung adenocarcinoma [1, 19]; however, AIS can have a solid component on CT, which makes difficult to make a correct diagnosis. With regard to surgery, lobectomy is considered for IAC. However, limited resection may be effective for AIS and MIA [1]. It is important to distinguish AIS or MIA from IAC for both management and treatment, as well as for predicting the prognosis. The previous study showed the correlation of solid portion on thin-section CT on the lung window setting and usual mediastinal setting, with pathologic invasiveness [23]. To further clarify the appropriate window setting that indicates pathological invasiveness, we measured the diameter of the solid component using four window settings: in addition to the routine lung and mediastinal window, two other window settings were used in this study.

Among these, the correlation coefficient of the routine mediastinal window setting (WW, 255 HU; WL, 50 HU) at our hospital was the largest. Other quantitative analyses of the solid component, including the CT values, the volume of the solid component, and the 50 th, 70 th, and 100 th percentiles showed correlations. The correlation coefficient of the 100 th percentile was the largest (*r* = 0.61).

Several previous studies have investigated the CT features associated with invasiveness in lung adenocarcinoma [4, 9, 12, 14, 19–23], although a few studies have focused on the solid component. Xu et al. reported that 52 of 218 part-solid nodules (23.85%) were pathologically proven to be AIS, and that smaller lesion size, well-defined border, and homogeneity of the solid component were significant independent predictors of AIS with alveolar collapse from IAC [14]. Our study showed that not only the diameter of the tumor and solid component but also the mean CT values, the volume of solid components, and the 50 th, 70 th, and 100 th percentiles were significantly larger in IAC than in AIS or MIA. The higher CT values of the solid component in IAC than those in AIS or MIA could be caused by differences in cell density, strength of alveolar collapse, and architectural distortion. A quantitative CT analysis focused on solid components would be useful for differentiating AIS from MIA and IAC.

Yanagawa et al. reported that a solid portion > 0.8 cm on the lung window setting or a solid portion > 0.6 cm on the mediastinal window setting on CT predicts histopathologic invasiveness [23]. Our study showed that the diameter of the solid component on the mediastinal window would be more useful than that on the lung window for indicating invasiveness, and the cut-off value was determined to be 0.8 cm; which is slightly larger than the previously reported value [23]. Other than stromal or vascular invasion, it has been reported that the solid component could correspond to a collapsed alveolar space, fibroblastic proliferation, infiltration, and mucin [13]. Our study included findings other than stromal or vascular invasion, although the correlation between the solid component on CT and pathological invasiveness was not assessed. In addition, the results may have been affected by the exclusion of cases with pure GGN from our study.

Other than the diameter of the solid component on CT, the volume of the solid component measured using CT software could also be useful for predicting IAC based on the results of our study. Automated calculations can be performed without intra- or inter-reader variability.

The present study was associated with some limitations. First, this was a retrospective study that analyzed a relatively small number of subjects. Prospective studies with larger sample sizes are needed to improve our research. Second, the correlation between the solid component on CT and pathological invasiveness was not assessed in this study.

In conclusion, we found that part-solid nodules were significantly more frequent in AIS and MIA than in IAC, considering lung adenocarcinoma with a solid component. Quantitative analyses, including both manual measurements and evaluation using CT software, are useful for differentiating between AIS or MIA and IAC.

## Data Availability

The datasets generated or analyzed during the study are available from the corresponding author on reasonable request.
